# Real-Time Single-Frequency GPS/MEMS-IMU Attitude Determination of Lightweight UAVs

**DOI:** 10.3390/s151026212

**Published:** 2015-10-16

**Authors:** Christian Eling, Lasse Klingbeil, Heiner Kuhlmann

**Affiliations:** Institute of Geodesy and Geoinformation, University of Bonn, Nussallee 17, 53115 Bonn, Germany; E-Mails: l.klingbeil@igg.uni-bonn.de (L.K.); heiner.kuhlmann@uni-bonn.de (H.K.)

**Keywords:** UAV, direct georeferencing, attitude determination, GPS, ambiguity resolution, MEMS IMU, magnetometer

## Abstract

In this paper, a newly-developed direct georeferencing system for the guidance, navigation and control of lightweight unmanned aerial vehicles (UAVs), having a weight limit of 5 kg and a size limit of 1.5 m, and for UAV-based surveying and remote sensing applications is presented. The system is intended to provide highly accurate positions and attitudes (better than 5 cm and 0.5${}^{\circ }$) in real time, using lightweight components. The main focus of this paper is on the attitude determination with the system. This attitude determination is based on an onboard single-frequency GPS baseline, MEMS (micro-electro-mechanical systems) inertial sensor readings, magnetic field observations and a 3D position measurement. All of this information is integrated in a sixteen-state error space Kalman filter. Special attention in the algorithm development is paid to the carrier phase ambiguity resolution of the single-frequency GPS baseline observations. We aim at a reliable and instantaneous ambiguity resolution, since the system is used in urban areas, where frequent losses of the GPS signal lock occur and the GPS measurement conditions are challenging. Flight tests and a comparison to a navigation-grade inertial navigation system illustrate the performance of the developed system in dynamic situations. Evaluations show that the accuracies of the system are 0.05${}^{\circ }$ for the roll and the pitch angle and 0.2${}^{\circ }$ for the yaw angle. The ambiguities of the single-frequency GPS baseline can be resolved instantaneously in more than 90% of the cases.

## 1. Introduction

The recent interest in unmanned aerial vehicles (UAVs) necessitates the development of small and lightweight direct georeferencing systems for the guidance, navigation and control (GNC) of UAVs and UAV-based surveying or remote sensing applications. Direct georeferencing comprises the determination of the position (e.g., *X*, *Y*, *Z*) and the attitude (e.g., *α*, *β*, *γ*) of a sensor or the vehicle relative to the Earth, represented in a predefined coordinate frame. The attitude is usually defined as the orientation between the body fixed coordinate frame and the navigation coordinate frame.

Although UAVs exist in many different size classes, this paper focuses on micro- and mini-sized UAVs, having a weight limit of 5 kg and a size limit of 1.5 m. Thus, the term UAV in this paper always relates to micro- and mini-sized UAVs.

In recent years, direct georeferencing systems have extensively been researched and used, especially in airborne applications [1,2]. However, these systems cannot easily be adopted for small UAVs, which is mainly due to weight and size constraints. Examples of the first approaches to the direct georeferencing of UAVs can be found in [3,4,5,6,7,8]. Nevertheless, in [3,4,5,6,7], direct georeferencing is not performed in hard real-time onboard the UAV. In particular, for the GNC and the realization of automatic operations, the real-time capability is very important. Falco *et al.* [8] describe the development of a real-time-capable low-cost system for the position and attitude determination of small UAVs. However, the system accuracies are only <2 m for the horizontal positions and <2${}^{\circ }$ for the yaw angle, which is not sufficient for many applications, e.g., surveying.

In this paper, a newly-developed direct georeferencing system is presented, which is intended to provide highly accurate positions and attitudes ($\sigma_{pos}$< 5 cm, $\sigma_{att}$<0.5${}^{\circ }$) in real-time onboard a UAV, using lightweight components. Even if the system estimates both positions and attitudes [9], this paper is focused on the attitude determination with the developed system. The algorithms are described in detail, and the accuracy and the performance of the attitude determination are comprehensively evaluated.

The challenge in the attitude determination of UAVs comes with the usage of small and lightweight IMUs (inertial measurement unit), such as micro-electro-mechanical system (MEMS) IMUs, since they have a significantly lower quality than the navigation-grade IMUs, which are usually applied in direct georeferencing systems. Without the frequent availability of absolute attitude information, the output of the gyroscopes of MEMS IMUs would be significantly wrong after a few seconds. This applies in particular to the yaw angle. While an IMU itself measures the gravity, which in the long term can be used to bound the roll and the pitch error, the yaw angle error is not bounded, if only IMU readings are available and no assumption about the motion behavior is made. To overcome this problem, there are multiple options, which may serve as a yaw reference for a UAV attitude determination.

One option comes with the availability of position information in situations where the UAV’s horizontal acceleration is not zero [10,11,12]. In this case, the yaw angle is correlated to the position observation. A drawback of this approach is clearly the need for non-zero accelerations.

Another option, which is often used in MEMS IMU-based attitude determination, is the integration of a magnetometer [12,13]. However, the main problem when using a magnetometer on a UAV is the distortion of the magnetic field measurements, caused by various time-constant and time-varying effects. Although most of the constant effects, coming from ferromagnetic material in the vicinity of the sensor, can be compensated by appropriate calibration methods [14,15], the non-constant effects, e.g., high electrical currents during the operation of rotary-wing UAVs, inhibit sub-degree attitude accuracies in most cases.

A third option is the realization of a GNSS multi-antenna system on the UAV to provide a direct yaw measurement. Examples for the use of multi-antenna GPS systems on lightweight UAVs can be found in [3,8,16]. Due to the low price and the small weight of some currently available single-frequency GPS receivers, this option is becoming more and more attractive. However, for the attitude determination, GPS carrier phases have to be observed, and a resolution of the carrier phase ambiguities is required. This is a challenging task, especially for low-cost single-frequency GPS observations [17]. During UAV applications in urban areas, frequent losses of the GPS signal lock can occur due to obstacles in the signal path. Since every loss of lock necessitates a re-initialization of the ambiguities, the ambiguity resolution process should be as fast as possible.

The contribution of this paper is the presentation and the evaluation of a small and lightweight real-time-capable direct georeferencing multisensor system, which also acts as an attitude and heading reference system (AHRS) for micro- and mini-sized UAVs. The desired attitude accuracy of the system is less than 0.5${}^{\circ }$ for all attitude angles. To achieve this and to overcome the disadvantages of the different yaw determination options, the following information is used and combined in a suitable way:

- 3D position measurement: This can be a code-based GPS position with accuracies of several meters or a cm-accurate RTK (real-time kinematic) GPS position. We use an RTK GPS position in this paper.
- MEMS IMU readings: The IMU readings include angular rates and accelerations, measured by three-axis gyroscopes and accelerometers.
- Single-frequency GPS observations: The GPS observations, measured by two onboard GPS receivers, enable the determination of an onboard GPS baseline, which contributes to the yaw determination.
- Magnetic field observations: The magnetic field observations come from a calibrated magnetometer, which is placed as far away as possible from the electric currents on the UAV platform.

In the following sections of this paper, we first explain the GPS attitude baseline determination algorithms, including the concept of an instantaneous ambiguity resolution for the single-frequency onboard GPS baseline. Afterwards, the details of the sensor integration and the system design will be presented. The evaluation of different field tests enables the assessment of the attitude accuracy and the ambiguity resolution performance of the system.

## 2. GPS Attitude Baseline Determination

It has been known for many years that GPS multi-antenna systems are well suited for the attitude determination of mobile objects [18,19]. The accuracy of a GPS attitude angle depends on the baseline length, which is limited to *ca.* 1 m for small UAVs, and the accuracy of the baseline coordinates. To achieve sub-cm accuracies of the baseline coordinates, which lead to sub-degree attitude accuracies for a 1-m baseline, carrier phases have to be observed, and the carrier phase ambiguities have to be fixed to their correct integer value. For kinematic applications, this ambiguity resolution should be fast and reliable at the same time.

In recent years, many different approaches have been investigated to allow for the ambiguity resolution in the attitude determination. For example, motion-based methods rely on the motion of the vehicle or changes in the receiver-satellite-geometry [19,20]. Many other procedures make use of the LAMBDA (Least-squares AMBiguity Decorrelation Adjustment) method [21]. Due to the integration of the known baseline length or geometry in the ambiguity objective function, this procedure has been improved for the attitude determination [22,23,24,25]. Further improvements are possible, when also inertial sensor readings are used to reduce the number of candidates in the ambiguity search space [16,26,27,28,29,30,31]. For example, in Eling *et al.* [29], an instantaneous ambiguity resolution procedure based on the ambiguity function method (AFM) [32], aided by MEMS gyroscopes, is presented. Roth *et al.* [28] applied an enhanced approach of the extended LAMBDA method, which has been developed by Mönikes *et al.* [16]. To accelerate the ambiguity resolution, they use the baseline length and magnetic field observations as constraints. However, as described in [31], a fast or even instantaneous ambiguity resolution with a constrained LAMBDA method can be difficult, when the ambiguity float solution is systematically wrong, due to poor GPS measurements under challenging GPS measurement conditions in urban areas.

In our approach, we aim for an instantaneous and reliable single-frequency GPS ambiguity resolution during UAV flights. Therefore, we use the information from additional sensors (GPS position, inertial sensors and magnetic field observations) and the known baseline length to improve the ambiguity float solution. Afterwards, we perform a search in the ambiguity domain, using the modified LAMBDA method (MLAMBDA) [33]. For a further search and also a validation step, we perform a transformation into the coordinate domain, where the AFM and further checks are also applied.

### 2.1. Overview

**Figure 1:**
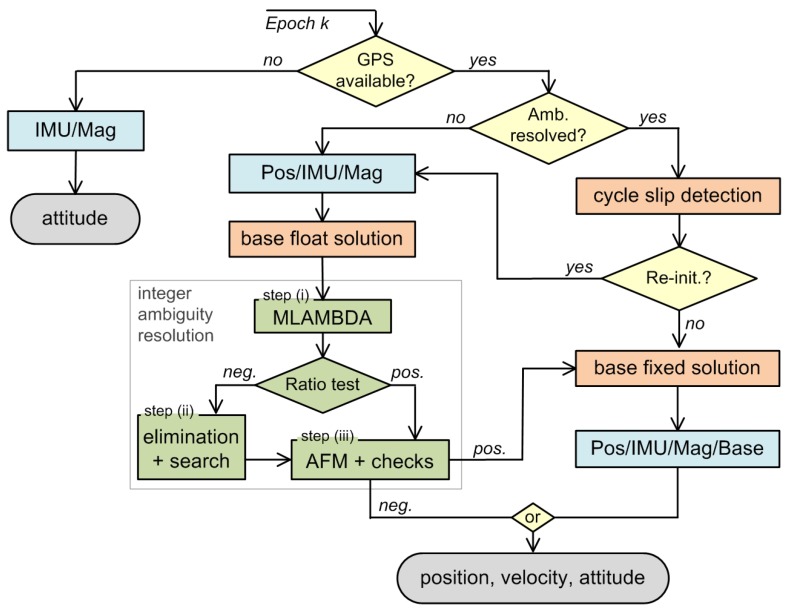
Flowchart of the attitude determination calculation steps. “Pos/IMU/Mag/Base” abbreviates a Kalman filtering, where a position measurement (Pos), IMU (inertial measurement unit) readings, magnetic field observations (Mag) and the GPS baseline (Base) are integrated. MLAMBDA is the modified LAMBDA method [33], and the AFM is the ambiguity function method [32].

Figure 1 shows the overall process of the attitude determination with the developed direct georeferencing system. To aid the ambiguity estimation of the GPS baseline, first, an approximate attitude is estimated in a Kalman filter (Pos/IMU/Mag), using a position measurement (Pos) (in our case, an RTK GPS position), IMU readings and magnetic field observations (Mag) (Section 3). Afterwards, these attitude results serve as an input to the GPS baseline determination, which consists of the base float solution and the integer ambiguity resolution (Section 2.2 and Section 2.3). If the ambiguities can be resolved successfully, the base fixed solution (Base) can also be integrated in the Kalman filter (Pos/IMU/Mag/Base).

The whole strategy assumes the availability of position measurements and GPS observations. If they are not available (no GPS observations), the sensor integration switches to a second mode (IMU/Mag), which only estimates the attitude based on inertial sensor and magnetometer readings (Section 4).

The coupling of the approximate information about the attitude and the GPS baseline raw data could generally also be realized in a tightly-coupled approach, but for reliability and practical reasons in the real-time programming, we decided to separate these steps here.

### 2.2. GPS Baseline Float Solution

In the first step of the ambiguity resolution, the ambiguities are estimated as real values in the float solution. This estimation is realized in a total state space extended Kalman filter (EKF). Beside the single-difference (SD) [34] ambiguities $N_{AB}^{j}$, the state vector $x_{SD}$ of this filter also contains the baseline parameters $\text{Δ}x_{b}^{e}$ and the first time derivative of the baseline parameters $\text{Δ}{\dot{x}}_{b}^{e}$:

(1) $$x_{SD}={\left[\begin{array}{ccc} \text{Δ}x_{b}^{e,T} & \text{Δ}{\dot{x}}_{b}^{e,T} & N_{AB}^{j,T} \end{array}\right]}^{T}$$

where *A* and *B* are the notations for both GPS antennas of the baseline and *j* is the notation for the satellites. The reason for estimating SD instead of double-difference (DD) [34] ambiguities is to avoid the hand over problem that would arise for DD ambiguities, when the reference satellite changes [35].

Measurement model: As observations DD carrier phases $\phi_{AB}^{jk}$ and DD pseudoranges $P_{AB}^{jk}$ on the GPS L1 frequency (L1=1575.42 MHz) are used:

(2) $$\begin{array}{cc} \phi_{AB}^{jk}\left(t_{k}\right) & =\frac{1}{\lambda }\rho_{AB}^{jk}\left(t_{k}\right)+\left(N_{AB}^{j}-N_{AB}^{k}\right)+\vartheta_{\phi } \end{array}$$

(3) $$\begin{array}{cc} P_{AB}^{jk}\left(t_{k}\right) & =\rho_{AB}^{jk}\left(t_{k}\right)+\vartheta_{P} \end{array}$$

where $\rho_{AB}^{jk}\left(t_{k}\right)$ is the geometric range DD, *λ* is the GPS L1 wavelength and *ϑ* is the noise.

To improve the ambiguity resolution, the prior information about the attitude, known from the Pos/IMU/Mag solution (Section 3), is added to the observation vector. For this, the known body-frame (b-frame) baseline parameters $\text{Δ}x_{b}^{b}$ are transformed into the e -frame (earth centered, earth fixed), using the $C_{b}^{e}$ rotation matrix, which represents the attitudes from the Pos/IMU/Mag solution:

(4) $$\text{Δ}{\bar{x}}_{b}^{e}=C_{b}^{e}\text{Δ}x_{b}^{b}$$

Finally, the known baseline length *s* can be added as a constraint to the observation vector. Accordingly, the observation vector reads:

(5) $$\begin{array}{cc} z_{k} & =\left[\begin{array}{cccccccccc} \phi_{AB}^{1k} & \ldots & \phi_{AB}^{mk} & P_{AB}^{1k} & \ldots & P_{AB}^{mk} & \text{Δ}{\bar{x}}_{b,x}^{e} & \text{Δ}{\bar{x}}_{b,y}^{e} & \text{Δ}{\bar{x}}_{b,z}^{e} & s \end{array}\right] \end{array}$$

A linearization of the measurement equations yields the measurement matrix:

(6) $$H_{k}=\left[\begin{array}{ccc} A_{n\times 3} & 0_{n\times 3} & \lambda D_{n\times m}\\ A_{n\times 3} & 0_{n\times 3} & 0_{n\times m}\\ I_{3\times 3} & 0_{3\times 3} & 0_{3\times m}\\ \frac{\text{Δ}x_{b}^{e,T}}{\left|\text{Δ}x_{b}^{e}\right|} & 0_{1\times 3} & 0_{1\times m} \end{array}\right],D=\left[\begin{array}{cccc} 1 & -1 & & \\ \vdots & & \ddots & \\ 1 & & & -1 \end{array}\right]$$

Therein, the matrix A includes the satellite/receiver geometry [34], and the matrix D is the double differencing matrix. n is the number of visible DD observations, and m=n+1 is the number of visible satellites.

As the stochastic model for the GPS observations, the following equation is used:

(7) $$\sigma_{\phi ,P}^{2}=2\left(a^{2}f_{\phi ,P}^{2}+{\left(b/\sin el\right)}^{2}f_{\phi ,P}^{2}\right)$$

where *f* is a factor, which is $f_{P}=100$ for the pseudoranges and $f_{\phi }=1$ for the carrier phases. el is the satellite elevation angle, and *a* and *b* are free parameters, weighting the influence of the elevation angle on the measurement noise. The choice of the values for *a* and *b* depends on various effects (antennas, receivers, antenna near-fields, *etc*.), which may be different for every measurement setup. In order to find realistic values representing the measurement noise of the GPS observations on our system during kinematic applications, we analyzed different flights. Based on the covariance matrices of the parameters and the residuals of the measurements, we found that a=2 mm and b=2 mm lead to the best results for the parameter estimation with our setup.

Finally, in the complete measurement noise matrix $R_{k}$, also the DD correlations are regarded:

(8) $$\begin{array}{cc} \Sigma_{\phi ,P} & =diag\left({\sigma_{\phi ,P}^{1}}^{2}\;\ldots \;{\sigma_{\phi ,P}^{m}}^{2}\right) \end{array}$$

(9) $$\begin{array}{cc} R_{k} & =\left[\begin{array}{cccc} D\Sigma_{\phi }D^{T} & 0 & 0 & 0\\ 0 & D\Sigma_{P}D^{T} & 0 & 0\\ 0 & 0 & \sigma_{\text{Δ}{\bar{x}}_{b}}I_{3\times 3} & 0\\ 0 & 0 & 0 & \sigma_{s} \end{array}\right] \end{array}$$

where $\sigma_{\text{Δ}{\bar{x}}_{b}}$ is the noise of the prior information and $\sigma_{s}$ the noise of the baseline length constraint.

System dynamics model: The standard total state space EKF time update is the following:

(10) $$\begin{array}{cc} x_{SD,k}^{-} & =\Psi_{k}x_{SD,k-1}^{+},\;\;P_{k}^{-}=\Phi_{k}P_{k-1}^{+}\Phi_{k}^{T}+G_{k}Q_{k-1}G_{k}^{T} \end{array}$$

We use a simple random walk model as the system dynamics model. Thus, the state transition matrix is composed of:

(11) $$\Psi_{k}=\left[\begin{array}{ccc} I_{3\times 3} & \text{Δ}t\;I_{3\times 3} & 0_{3\times m}\\ 0_{3\times 3} & I_{3\times 3} & 0_{3\times m}\\ 0_{m\times 3} & 0_{m\times 3} & I_{m\times m} \end{array}\right]$$

and $G_{k}$ is given by:

(12) $$G_{k}=\left[\begin{array}{cc} \frac{1}{2}\text{Δ}t^{2}\;I_{3\times 3} & 0_{3\times m}\\ \text{Δ}t\;I_{3\times 3} & 0_{3\times m}\\ 0_{m\times 3} & \text{Δ}t\;I_{m\times m} \end{array}\right]$$

The process noise matrix $Q_{k}$ is a diagonal matrix consisting of two types of process noise. The process noise for the first time derivative of the baseline parameters is set to $\sigma_{\text{Δ}\dot{x}}$ = 1 m/s. This choice reflects the approximate dynamic capabilities of the UAV, but does not smooth the baseline parameters too much. In contrast, the ambiguity parameters are assumed to be constant. However, to allow for a better reaction of the ambiguity parameters on changing satellite configurations, the noise parameter for the ambiguities is not set to zero, but to a very small value: $\sigma_{N}=1\cdot 10^{-4}$ cycles.

(13) $$Q_{k}=diag\left(\sigma_{\text{Δ}\dot{x}}^{2}\;\sigma_{\text{Δ}\dot{x}}^{2}\;\sigma_{\text{Δ}\dot{x}}^{2}\;{\sigma_{N}^{1}}^{2}\;\ldots \;{\sigma_{N}^{m}}^{2}\right)$$

Since the initialization of the ambiguity parameters, which is based on the difference between the SD pseudoranges and the SD carrier phases, is very imprecise, the process noise for new initialized ambiguities is set to a very large value of $\sigma_{N}$ = 100 cycles. In this way, new initialized ambiguities do not have any influence on the estimation of the other parameters, but benefit from the current float solution.

In the final step of the float solution, the estimated SD float ambiguities and their covariance matrix have to be transformed to DD values, applying the D-matrix again.

### 2.3. Integer Ambiguity Resolution

The results of the float solution are real-valued DD ambiguities and their covariance matrix. However, the true ambiguities are integer valued, and they need to be found within the solution space, which is spanned by the estimated float ambiguities and their covariances. This process is basically a search problem, called integer ambiguity resolution, and it is the key to enable mm-accuracies for the baseline parameters. In our approach, three steps are performed (see also Figure 1) to resolve the ambiguities correctly:

Step (i): In the first step of the integer ambiguity resolution, the MLAMBDA method [33] is applied. The MLAMBDA method is an ambiguity search technique in the ambiguity domain and an extension of the well-known LAMBDA method [21], leading to faster computation times [33]. The MLAMBDA method is based on an integer least squares problem to obtain the estimates of the DD integer ambiguities solving the minimization problem [21]:

(14) $$min{\left(\hat{N}-N\right)}^{T}Q_{\hat{N}}^{-1}\left(\hat{N}-N\right)\;\;\;\text{with}\;N\in Z^{n}$$

where N is the integer ambiguity vector, $\hat{N}$ is the float ambiguity vector and $Q_{\hat{N}}$ represents its covariance matrix. The inputs for the MLAMBDA method are the DD float ambiguities and their covariance matrix. Due to the aid of the additional information about the attitude, known from the Pos/IMU/Mag solution (Section 3), the baseline float solution is more accurate, compared to a GPS-only float solution. The more accurate the float ambiguities are, the more likely is a fast and correct ambiguity resolution with the MLAMBDA method. Thus, there is a good chance that the ambiguities can already be fixed at this step of the ambiguity resolution process in our approach.

As outputs of the MLAMBDA method, we consider ten sets of ambiguity candidates. To make a decision if any of these sets can be fixed, we first apply the simple ratio test [36], where the squared norm of the ambiguity residuals of the best set of ambiguities ($R_{1}$) and the second best set of ambiguities ($R_{2}$) is compared. Only if the quotient $R_{2}/R_{1}$ exceeds a threshold of three, the ambiguities of the best set will be fixed to integer values.

Step (ii): In case the ambiguities could not be fixed within Step (i), further effort is needed. The ten best solutions from the MLAMBDA method are considered further. In the first instance, we try to perform an elimination of single ambiguity parameters, which probably prevent the fixing of all of the other ambiguities. For example, in the case that all ten sets of ambiguities are equal except the ambiguity of one DD observation, this one varying ambiguity parameter is omitted. In the case that more than one ambiguity parameter is varying in the ten sets of ambiguities, always one ambiguity parameter is eliminated in a random order, and the MLAMBDA method is applied again, until the ratio test is positive or a maximum number of iterations is reached. Due to the elimination of single ambiguity parameters, some of the ten sets of ambiguity candidates can be omitted now, since they probably became equal to other ambiguity sets.

Step (iii): In the last step of the ambiguity estimation, a transformation into the coordinate domain is performed. This means that all of the remaining sets of ambiguity candidates are used to determine baseline parameters. Afterwards, these baseline parameters are tested in the ambiguity function method (AFM) [32], which is a search technique based on a trigonometric cost function, the ambiguity resolution function (ARF):

(15) $$\text{ARF}\left(b_{x},b_{y},b_{z}\right)=\sum_{j=1}^{n}cos2\pi \left[\phi_{obs}^{jk}-\phi_{calc}^{jk}\left(b_{x},b_{y},b_{z}\right)\right]$$

By use of the determined baseline parameters, all observed DD carrier phases $\phi_{obs}^{jk}$ can also be calculated ($\phi_{calc}^{jk}$). If the determined baseline parameters are correct, the differences between the observed and the calculated DD carrier phases are the integer ambiguities. Therefore, the result of the ARF should be close to the number of the available DD carrier phases (*n*) if the correct candidate is tested. Beside the AFM, we also consider the lengths and the residuals of the determined baseline parameters to check the correctness of the ambiguities.

As can be seen in Figure 1 Step (iii) of the ambiguity resolution process is also applied to fixed solutions from Step (i) or (ii), to validate these results. Thus, due to the combination of Steps (i), (ii) and (iii), a fast and reliable ambiguity resolution can be performed at the same time.

### 2.4. Fixed Solution

In the fixed solution, the final baseline parameters $\text{Δ}x_{b}^{e}$ are estimated. Once the ambiguities have been fixed successfully, they can generally be held fixed for the further epochs, as long as no signal interruption or cycle slip occurs. To ensure that the ambiguities are held fixed correctly, they are resolved independently, epoch by epoch, until the same ambiguity resolution has been determined in ten consecutive epochs. The risk that the same incorrect ambiguity resolution is found in ten consecutive epochs during dynamic applications is negligible.

### 2.5. Cycle Slip Detection

A loss of a satellite signal lock leads to a reinitialization of the corresponding integer counter in the GPS receiver. As a result, the previously known ambiguity parameter of the affected satellite is not valid any longer. These cycle slips have to be detected, if the ambiguities are held fixed. The cycle slip detection can be done using a Kalman filter [37,38], where an exceptional change in the carrier phases between two measurement epochs will be conspicuous, when a good prediction of the baseline parameters is available. These requirements are met here, since the motion behavior of the baseline parameters between two epochs is well known from the sensor integration.

The transfer of the information from the sensor integration to the cycle slip detection is realized using the attitude change, which can be converted into the first deviation of the baseline parameters $\text{Δ}{\tilde{\dot{x}}}_{b}^{e}$. Finally, this information serves as control input, to check the consistency of the ambiguity parameters in a second GPS Kalman filter, which is decoupled from the float solution.

The state vector $x_{C}$ of this simple filter includes the baseline and the ambiguity parameters. The system dynamics model is a random walk model:

(16) $$\begin{array}{cc} x_{C,k}^{-} & =\Psi_{k}x_{C,k-1}^{+}+B_{k}u_{k}\\ & =\left[\begin{array}{cc} I_{3\times 3} & 0_{3\times m}\\ 0_{m\times 3} & I_{m\times m} \end{array}\right]\left[\begin{array}{c} \text{Δ}x_{b}^{e}\\ N_{AB}^{j} \end{array}\right]+\left[\begin{array}{c} \text{Δ}t\;I_{3\times 3}\\ 0_{m\times 3} \end{array}\right]\text{Δ}{\tilde{\dot{x}}}_{b}^{e} \end{array}$$

The observation vector includes the carrier phases and the pseudoranges:

(17) $$z_{k}={\left[\begin{array}{cccccc} \phi_{AB}^{1k} & \ldots & \phi_{AB}^{mk} & P_{AB}^{1k} & \ldots & P_{AB}^{mk} \end{array}\right]}^{T}$$

and the measurement matrix represents the nonlinear functional model for a single baseline:

(18) $$H_{k}=\left[\begin{array}{cc} A_{n\times 3} & \lambda D_{n\times m}\\ A_{n\times 3} & 0_{n\times m} \end{array}\right]$$

Cycle slips can now be detected considering the innovation:

(19) $$d_{k}=z_{k}-H_{k}x_{C,k}^{-}$$

In case no cycle slips exist, the innovation of the carrier phases remains close to zero. When the innovation of one DD carrier phase exceeds a threshold d>0.1 m, a cycle slip is probably the reason, and the corresponding ambiguity parameter is re-initialized. If at least three DD carrier phases show inaccuracies, the complete ambiguity set is rejected, and the ambiguity resolution process is re-initialized.

## 3. The Sensor Integration

The sensor integration can be separated into the strapdown algorithm (SDA) and the Kalman filter update. In the SDA, the highly dynamic movement of the system is determined integrating the angular rates and accelerations of the MEMS IMU in real time. As a result of various errors (initial alignment, inertial sensors, computational, *etc*.), the SDA solution is drifting over time. Therefore, the additional sensors, providing long-term, stable measurements, are needed to correct and bound the drift of the inertial sensor integration [39]. The estimation of the SDA errors is realized in an error state space Kalman filter. Some advantages for this approach can, for example, be found in [40,41].

In the following, the SDA and the Kalman filter update will be summarized. More details can be found in [42,43,44]. Here, the navigation equations of the b-frame are expressed in the e-frame. The b-frame to e-frame rotation $C_{b}^{e}$ is split into a rotation of the b-frame with respect to the local-level navigation frame (n-frame) $C_{b}^{n}$ and a rotation of the n-frame with respect to the e-frame $C_{n}^{e}$. Since the axes of the n-frame are aligned with the directions east, north and up, the $C_{n}^{e}$ rotation is:

(20) $$C_{n}^{e}=\left[\begin{array}{ccc} -\sin\lambda_{o} & -\sin\varphi_{o}\cos\lambda_{o} & \cos\varphi_{o}\cos\lambda_{o}\\ \cos\lambda_{o} & -\sin\varphi_{o}\sin\lambda_{o} & \cos\varphi_{o}\sin\lambda_{o}\\ 0 & cos\varphi_{o} & \sin\varphi_{o} \end{array}\right]$$

where $\lambda_{o}$ and $\varphi_{o}$ are the ellipsoidal coordinates of the b-frame origin.

The rotation from the b-frame to the n-frame may be expressed as the product of three separate rotations:

(21) $$C_{b}^{n}=C_{n}^{b,T}=R_{3}{\left(\gamma \right)}^{T}\cdot R_{2}{\left(\beta \right)}^{T}\cdot R_{1}{\left(\alpha \right)}^{T}$$

using the Euler angles roll *α*, pitch *β* and yaw *γ*.

In the full state vector x, not the Euler angles, but a quaternion q is used for the attitude representation. Further states are the position $x_{p}^{e}$, the velocity $v_{eb}^{e}$, the accelerometer bias $b_{a}^{b}$ and the gyro bias $b_{\omega }^{b}$:

(22) $$x={\left[\begin{array}{ccccc} x_{p}^{e,T} & v_{eb}^{e,T} & q^{T} & b_{a}^{b,T} & b_{\omega }^{b,T} \end{array}\right]}^{T}$$

### 3.1. Strapdown Algorithm

Within the SDA, the MEMS IMU readings are used to propagate the attitude, the velocity and the position of the system.

Attitude: The gyroscopes of the IMU measure the attitude change $\omega_{ib}^{b}$ of the b-frame with respect to an inertial frame represented in the b-frame. Since the Earth rotation rate $\omega_{ie}^{e}$ is small compared to the measurement noise of the MEMS gyroscopes, the approximation:

(23) $$\omega_{eb}^{b}\approx \omega_{ib}^{b}$$

is valid. Assuming that the direction of the angular rate vector $\omega_{eb}^{b}$ remains fixed in space during the time interval $\text{Δ}t=t_{k}-t_{k-1}$, the rotation angle $\text{Δ}\sigma_{k}$ can be determined with the trapezoidal rule:

(24) $$\text{Δ}\sigma_{k}\approx \left(\frac{\omega_{eb,k-1}^{b}+\omega_{eb,k}^{b}}{2}\right)\text{Δ}t$$

With the quaternion $\text{Δ}q_{k}$:

(25) $$\text{Δ}q_{k}=\left[\begin{array}{c} \cos(\left|\text{Δ}\sigma_{k}\right|/2)\\ a_{s}\text{Δ}\sigma_{x,k}\\ a_{s}\text{Δ}\sigma_{y,k}\\ a_{s}\text{Δ}\sigma_{z,k} \end{array}\right],\;\;a_{s}=\frac{\sin(\left|\text{Δ}\sigma_{k}\right|/2)}{\left|\text{Δ}\sigma_{k}\right|}$$

the current attitude $q_{k}$ results from:

(26) $$q_{k}=q_{k-1}\text{Δ}q_{k}$$

where $q_{k}$ represents the quaternion relating the b-frame to e-frame axes at the epoch *k*.

Velocity: The velocity propagation is based on the integration of the measured accelerations $a_{ib}^{b}$:

(27) $${\dot{v}}_{eb}^{e}=C_{b}^{e}a_{ib}^{b}-2\omega_{ie}^{n}\times v_{eb}^{e}+g_{l}^{e}$$

The assumption of a constant direction of the acceleration vector during the time interval Δt is also valid, with the result that Equation (27) can be simplified:

(28) $${\dot{v}}_{eb}^{e}\approx C_{b}^{e}a_{ib}^{b}+g_{l}^{e}$$

Then, the velocity at time *k* can be determined applying Equation (29).

(29) $$v_{eb,k}^{e}=v_{eb,k-1}^{e}+C_{b}^{e}\left[a_{ib}^{b}+\frac{1}{2}\text{Δ}\sigma_{k}\times a_{ib}^{b}\right]\text{Δ}t+g_{l}^{e}\text{Δ}t$$

The vector $g_{l}^{e}$ is the local gravitation vector represented in the e-frame.

Position: Finally, the current position may be derived integrating the velocity. For this integration, again, the trapezoidal rule is used, since the integration time is short here:

(30) $$x_{p,k}^{e}=x_{p,k-1}^{e}+\left(\frac{v_{eb,k-1}^{e}+v_{eb,k}^{e}}{2}\right)\text{Δ}t$$

### 3.2. Kalman Filter Update

The Kalman filter update is based on an error state extended Kalman filter, which means that the estimated state is not the full state vector itself, as described in Equation (22), but the error vector between the SDA predicted state and the full state. To estimate this error, the following measurements are used:

- the GPS position (Pos) ${\tilde{x}}_{a}^{e}$ of the GPS antenna reference point, expressed in the e-frame,
- the GPS attitude baseline vector (Base) $\text{Δ}{\tilde{x}}_{b}^{e}$, expressed in the e-frame and,
- the magnetic field vector (Mag) ${\tilde{h}}^{b}$, measured in the b-frame.

The error state vector δx includes the error vectors for the position $\delta x_{p}^{e}$, the velocity $\delta v_{eb}^{e}$, the attitude δψ, the accelerometer bias $\delta b_{a}$ and the gyro bias $\delta b_{\omega }$:

(31) $$\delta x=\left[\begin{array}{ccccc} \delta x_{p}^{e,T} & \delta v_{eb}^{e,T} & \delta \psi^{T} & \delta b_{a}^{b,T} & \delta b_{\omega }^{b,T} \end{array}\right]$$

System dynamics model: The derivation of the error dynamics equations can, for example, be found in [42] or [45]. Hence, the linearized continuous system dynamics model specialized to the e-frame is:

$$\begin{array}{cc} \dot{\delta x} & =F\delta x+Gn\\ & =\left[\begin{array}{ccccc} 0 & I & 0 & 0 & 0\\ 0 & 0 & -\left[{\hat{a}}_{ib}^{e}\times \right] & -{\hat{C}}_{b}^{e} & 0\\ 0 & 0 & 0 & 0 & -{\hat{C}}_{b}^{e}\\ 0 & 0 & 0 & 0 & 0\\ 0 & 0 & 0 & 0 & 0 \end{array}\right]\left[\begin{array}{c} \delta x_{p}^{e}\\ \delta v_{eb}^{e}\\ \delta \psi \\ \delta b_{a}^{b}\\ \delta b_{\omega }^{b} \end{array}\right]+\left[\begin{array}{cccc} 0 & 0 & 0 & 0\\ {\hat{C}}_{b}^{e} & 0 & 0 & 0\\ 0 & {\hat{C}}_{b}^{e} & 0 & 0\\ 0 & 0 & I & 0\\ 0 & 0 & 0 & I \end{array}\right]\left[\begin{array}{c} n_{a}\\ n_{\omega }\\ {n_{b}}_{a}\\ {n_{b}}_{\omega } \end{array}\right] \end{array}$$

where 0 is always a 3 × 3 zero matrix and I a 3 × 3 identity matrix. $\left[{\hat{a}}_{ib}^{e}\times \right]$ denotes a 3 × 3 skew symmetric matrix with elements of the bias-corrected acceleration vector $a_{ib}^{b}$, which has been rotated into the e-frame. The vector n includes the stochastic variables.

Measurement model: For the integration of the GPS positions the lever arm $l^{b}$ between the GPS antenna reference point $x_{a}^{e}$ and the system reference point $x_{p}^{e}$, which can be identical to the IMU reference point, has to be regarded [45]:

(32) $$\begin{array}{cc} {\tilde{x}}_{a}^{e} & =x_{p}^{e}+C_{b}^{e}l^{b}+\vartheta_{x_{a}}=x_{p}^{e}+\left(I+\Psi \right){\hat{C}}_{b}^{e}l^{b}+\vartheta_{x_{a}}\\ & =x_{p}^{e}+\left(I+\Psi \right){\hat{l}}^{e}+\vartheta_{x_{a}}=x_{p}^{e}+{\hat{l}}^{e}-\left[{\hat{l}}^{e}\times \right]\psi +\vartheta_{x_{a}} \end{array}$$

where $\vartheta_{x_{a}}$ denotes the GPS position measurement noise.

Due to the coupling between the GPS position and the measured accelerations from the IMU, the yaw angle error is bounded in the presence of horizontal accelerations. However, in the absence of horizontal accelerations, for example when the UAV is hovering, the yaw angle is still unobserved [12].

To overcome the problem of an undetermined yaw angle, the GPS attitude baseline is arranged on the UAV. In the measurement model, the link between the GPS baseline and the attitude determination is given by the transformation of the b-frame GPS baseline coordinates $\text{Δ}x_{b}^{b}$, which can be determined by calibration measurements, into the e-frame:

(33) $$\begin{array}{cc} \text{Δ}{\tilde{x}}_{b}^{e} & =C_{b}^{e}\text{Δ}x_{b}^{b}+\vartheta_{x_{b}}=\left(I+\Psi \right){\hat{C}}_{b}^{e}\text{Δ}x_{b}^{b}+\vartheta_{x_{b}}\\ & =\left(I+\Psi \right)\text{Δ}{\hat{x}}_{b}^{e}+\vartheta_{x_{b}}=\text{Δ}{\hat{x}}_{b}^{e}-\left[\text{Δ}{\hat{x}}_{b}^{e}\times \right]\psi +\vartheta_{x_{b}} \end{array}$$

where $\vartheta_{x_{b}}$ is the measurement noise of the GPS attitude baseline.

Finally, as long as the ambiguities of the GPS baseline are unresolved, the baseline parameters do not have the quality to correct the yaw error adequately. To be able to also bound the yaw error in these cases, we integrated magnetic field sensors into our system.

Magnetic field sensors, when used as a compass, measure two or three components of the Earth’s magnetic field in the sensor system. Therefore, they enable the determination of the orientation of the sensor system relative to the Magnetic North Pole. However, the main difficulties with magnetic field sensors are their sensitivities to ferromagnetic material in the vicinity of the sensor. In case this material is part of the sensor platform, disturbances can be compensated by calibration [15]. Nevertheless, the changing currents of the rotors or ferromagnetic material in the environment can still lead to distortions of the magnetic field observations. This is why the attitude accuracy, based on magnetometer measurements, is usually only in the range of a few degrees, which is still good enough to bound the gyro drift.

The measurement equation for a magnetic field measurement ${\tilde{h}}^{b}$ is the following [12]:

(34) $$\begin{array}{cc} {\tilde{h}}^{b} & =C_{b}^{e,T}h^{e}+\vartheta_{m}={\hat{C}}_{b}^{e,T}\left(I-\Psi \right)h^{e}+\vartheta_{m}\\ & ={\hat{C}}_{b}^{e,T}h^{e}-{\hat{C}}_{b}^{e,T}\Psi h^{e}+\vartheta_{m}={\hat{C}}_{b}^{e,T}h^{e}+{\hat{C}}_{b}^{e,T}\left[h^{e}\times \right]\psi +\vartheta_{m} \end{array}$$

where $h^{e}$ is the known local magnetic field vector, represented in the e-frame, and $\vartheta_{m}$ is the magnetometer measurement noise.

Since erroneous magnetic field observations can lead to significant systematic attitude errors in all three axis, Equation (35) is rearranged, with the result that the magnetic field observations only have an impact on the yaw angle determination:

(35) $$\begin{array}{c} {\tilde{h}}^{b}={\hat{C}}_{b}^{e,T}h^{e}+{\hat{C}}_{b}^{e,T}\left[\begin{array}{ccc} 0 & 0 & {\hat{h}}_{y}^{e}\\ 0 & 0 & -{\hat{h}}_{x}^{e}\\ 0 & 0 & 0 \end{array}\right]\psi +\vartheta_{m} \end{array}$$

With the ambiguities fixed, the parameters of the baseline can be determined with a mm-accuracy. For a baseline with a length of 1 m, this leads to attitude accuracies of approximately 0.2–0.5${}^{\circ }$ for the yaw angle. Since the roll and pitch angle can be determined more reliably regarding the relation to the gravitation vector, the GPS baseline should also only be used to estimate the yaw angle error:

(36) $$\text{Δ}{\tilde{x}}_{b}^{e}=\text{Δ}{\hat{x}}_{b}^{e}-\left[\begin{array}{ccc} 0 & 0 & \text{Δ}{\hat{x}}_{b,y}^{e}\\ 0 & 0 & -\text{Δ}{\hat{x}}_{b,x}^{e}\\ 0 & 0 & 0 \end{array}\right]\psi +\vartheta_{x_{b}}$$

In case the ambiguities of the GPS baseline are unresolved so far, only the GPS positions and the magnetic field observations are used in the Pos/IMU/Mag integration (Figure 1), which serves as the prior information for the GPS baseline ambiguity resolution. If the ambiguities are resolved successfully, the Pos/IMU/Mag/Base solution can be determined, where all available sensory input is integrated.

Kalman filter formulation: In the prediction step of the Kalman filter, only the error covariance matrix is propagated:

(37) $$P_{k}^{-}=\Phi_{k}P_{k-1}^{+}\Phi_{k}^{T}+G_{k}Q_{k-1}G_{k}^{T}$$

Therein, the matrices $\Phi_{k}$ and $G_{k}$ are the discretizations of the matrices F and G at the time *k*. The matrix $Q_{k-1}$ includes the discrete time noise.

The filter update is computed using the standard Kalman filter equations:

(38) $$\begin{array}{cc} K_{k} & =P_{k}^{-}H_{k}^{T}{\left(H_{k}P_{k}^{-}H_{k}^{T}+R_{k}\right)}^{-1} \end{array}$$

(39) $$\begin{array}{cc} P_{k}^{+} & =\left(I-K_{k}H_{k}\right)P_{k}^{-} \end{array}$$

(40) $$\begin{array}{cc} \delta x & =K_{k}\left(z_{k}-y_{k}\right) \end{array}$$

where $K_{k}$ is the Kalman gain matrix, $R_{k}$ is the measurement noise matrix, $H_{k}$ is the measurement matrix, $z_{k}$ is the observation vector and $y_{k}$ is the vector of the predicted observations, which are based on the results of the SDA. For the update of the full state vector, the error state vector is simply added to the SDA-derived state vector. For the attitude update, Equation (25) and Equation (26) have to be applied using δψ instead of Δσ.

## 4. Special Case: GPS Unavailability

When GPS observations have not been available for a few seconds, the Kalman filter, as it was presented in Section 3, cannot be applied any longer. The reasons for this are that the position and velocity errors, resulting from the inertial sensor integration, cannot be bounded without the GPS observations. Furthermore, the accelerometer biases are no longer observable longer. On this account, the system is intended to stop providing position and velocity estimates, and the Kalman filter update switches to a second mode [12], which is referred to as the IMU/Mag solution in Figure 1.

The Kalman filter in the second mode is a subset of the previously-presented filter (Section 3). The attitude prediction is still determined integrating the angular rates, but the state vector only consists of the attitude and the gyro bias errors. Since the magnetic field observations are not enough information to observe the full orientation (the rotation around the Earth’s magnetic field axis would be undetermined), the accelerometer readings are added to the measurement model. The measurement equation for the accelerations is the following [45]:

(41) $$\begin{array}{cc} {\tilde{a}}_{ib}^{b} & =-C_{b}^{e,T}g_{l}^{e}+\vartheta_{a}=-{\hat{C}}_{b}^{e,T}\left(I-\Psi \right)g_{l}^{e}+\vartheta_{a}\\ & =-{\hat{C}}_{b}^{e,T}g_{l}^{e}-{\hat{C}}_{b}^{e,T}\left[g_{l}^{e}\times \right]\psi +\vartheta_{a} \end{array}$$

It is assumed that the main force measured by the accelerometers is the local Earth gravitation vector $g_{l}^{e}$. The influence of the trajectory dynamics on the accelerometer measurements is modeled by a large measurement noise with $\vartheta_{a}$ = 3 m/s${}^{2}$.

## 5. System Realization

**Figure 2:**
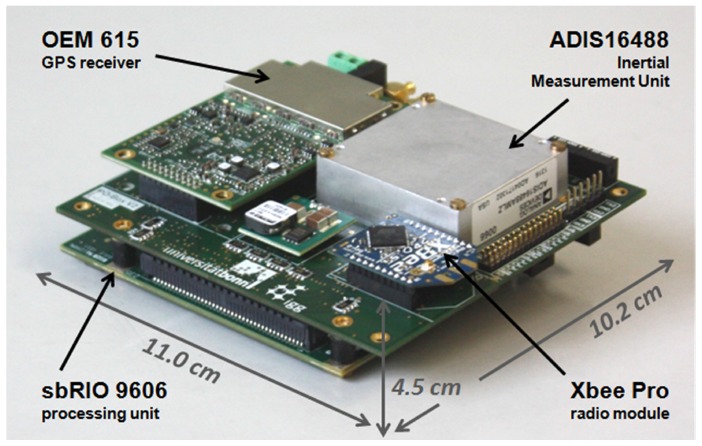
The direct georeferencing system, with its components.

The developed direct georeferencing system is shown in Figure 2. Its dimensions are 11.0 cm × 10.2 cm × 4.5 cm, and its weight is 240 g, without the GPS antennas.

The system consists of a dual-frequency geodetic-grade GPS receiver (Novatel OEM 615), which acts as the main positioning device. Together with the GPS raw data of a master station, which is transmitted via a radio module (XBee Pro 868), it allows for an RTK GPS (real-time kinematic) positioning with a rate of 10 Hz, leading to centimeter position accuracies.

The dual-frequency GPS receiver also serves as one receiver of the onboard GPS baseline for the GPS attitude determination (1 Hz). However, for the attitude determination, only the L1 GPS frequency is used, since the second onboard GPS device is a low-cost single-frequency GPS receiver (Ublox LEA6T).

The tactical grade IMU (Analog Devices Adis16488), including three-axis gyroscopes and accelerometers, provides high rate inertial data (100 Hz), which is used for the position and attitude determination. A magnetometer (Honeywell HMC5883L) is placed at the outer end of one of the rotor-free UAV arms, where it is not affected by electric currents.

A real-time processing unit (National Instruments sbRIO 9606), which is a reconfigurable IO board, including an FPGA (field programmable gate array) and a 400-MHz processor, enables the onboard position and attitude determination in real time.

**Figure 3:**
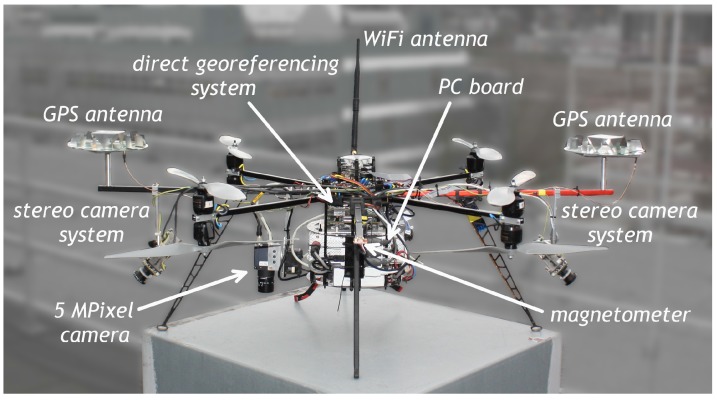
The UAV platform, equipped with different sensors and systems.

Figure 3 shows the current version of the UAV platform, which has been developed within a project called Mapping on Demand [46]. In this project, the UAV is intended to fly fully autonomously. Therefore, additional sensors, such as two stereo camera pairs and a 5 MPixel camera, are also fixed on the platform.

The GPS antennas of the attitude baseline can be seen on the left and the right side of Figure 3. The baseline length is 92 cm. Both antennas are geodetic-grade antennas (3G + C, navXperience), which are originally too heavy for UAV applications. In order to reduce weight, we omitted all unnecessary components of the antennas, such as the antenna housings and the 5/8” screw threads. Due to the changes in the antenna design, we had to recalibrate the antennas in our anechoic chamber [9,47]. By comparison to the original antenna, the antenna modifications led to significant changes in the phase center offsets (approximately 4 cm in the up, <1 mm in the north and east component) and in the phase center variations (<5 mm) of the antennas.

## 6. Results

**Figure 4:**
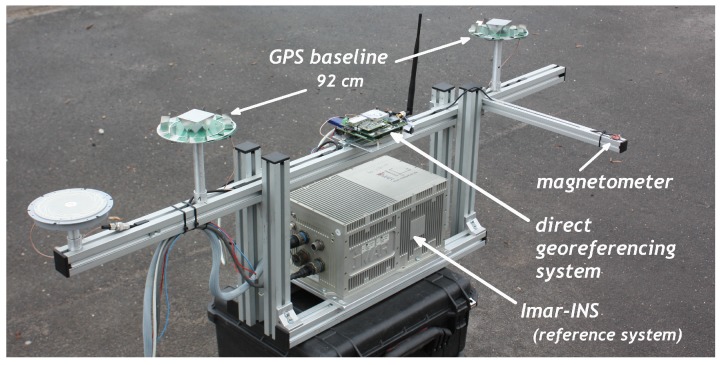
The measurement setup for the accuracy evaluation tests. This setup replicates the direct georeferencing of the UAV platform and includes a reference system for the attitude accuracy evaluations.

In this section, the ambiguity resolution performance and the attitude accuracy of the direct georeferencing system will be evaluated. For the evaluation of the ambiguity resolution, real flight test data are used. Since it is difficult to get a highly accurate attitude reference for UAV flights, a test setup has been applied for the accuracy evaluation. On this setup (Figure 4), the direct georeferencing system, the GPS antennas and the magnetometer are mounted in the same configuration as on the UAV platform. As the reference system, a navigation-grade INS (inertial navigation system) from Imar has been used, which consists of fiber optical gyros (FOG) and provides attitudes with a rate of 1000 Hz. The specifications of the Imar INS are shown in Table 1, together with the specifications of the MEMS IMU (Adis16488), which is used on the direct georeferencing system. Due to its high level of bias stability (<0.003–0.01${}^{\circ }$/h) and the low influence of the angular random walk (0.001${}^{\circ }$/$\sqrt{h}$), the attitudes of the Imar INS serve as an excellent attitude reference for about one hour after the measurement start.

**Table 1:** Specifications of the IMUs.

Specifications	Adis16488		Imar-INS _(iNAV-FJI-LSURV)_
	Gyros	Accelerometer	Fibre Optic Gyros
Full Scale	±450${}^{\circ }$/s	±18 g	±150${}^{\circ }$/s
Bias Stability	6${}^{\circ }$/h	0.1 mg	<0.003–0.01${}^{\circ }$/h
Random Walk	0.3${}^{\circ }$/$\sqrt{h}$	0.029 m/s/$\sqrt{h}$	0.001${}^{\circ }$/$\sqrt{h}$

### 6.1. Ambiguity Resolution

Concerning the evaluation of the ambiguity resolution, there are two questions we would like to answer. To make this possible, data of three flight tests (Flights 1–3) and three measurements with the previously-presented test setup (Tests 1–3) are used. All of these measurements were performed on different days and at different locations.

1. How often does the ambiguity resolution algorithm succeed in fixing the ambiguities in the first epoch (instantaneously) after a loss of lock?

Table 2 shows single-epoch ambiguity resolution success rates. For calculating these values, the complete integer ambiguity estimation process was re-initialized in every single epoch of the datasets. In the presentation of these values, a distinction is made between the three steps (Steps i–iii) of our ambiguity resolution algorithm (Section 2.3), on the one hand, and a standard ambiguity resolution algorithm, on the other hand. This standard ambiguity resolution algorithm includes a float solution without any prior information, an integer ambiguity estimation with the MLAMBDA method and a ratio test with a threshold of three.

**Table 2:** Single-epoch ambiguity resolution success rates.

	Standard (%)	Step (i) (%)	Step (ii) (%)	Step (iii) (%)
Flight 1	9.85	83.25	97.79	96.47
Flight 2	7.28	98.06	98.66	99.11
Flight 3	4.19	78.87	92.61	93.48
Test 1	24.92	95.95	98.75	99.53
Test 2	34.84	84.70	91.21	91.78
Test 3	32.65	84.69	96.17	96.17

It can be seen that our algorithm leads to instantaneous ambiguity resolutions in more than 90% of the epochs. Compared to the standard approach, this is a significant improvement, especially during the UAV flights, where the GPS measurement conditions have been more challenging, due to buildings and vegetation in the surrounding areas.

Within the different steps of our algorithm, a significant increase of the success rate can be seen between Step (i) and Step (ii), whereas the difference between Step (ii) and Step (iii) is smaller for the single-epoch ambiguity resolutions. In Flight 1, the success rate after Step (iii) even is smaller than after Step (ii). The reason for this is that some fixed solutions from Step (i) or (ii) have been rejected in the validation of Step (iii).

2. What is the average time to fix the ambiguities?

**Figure 5:**
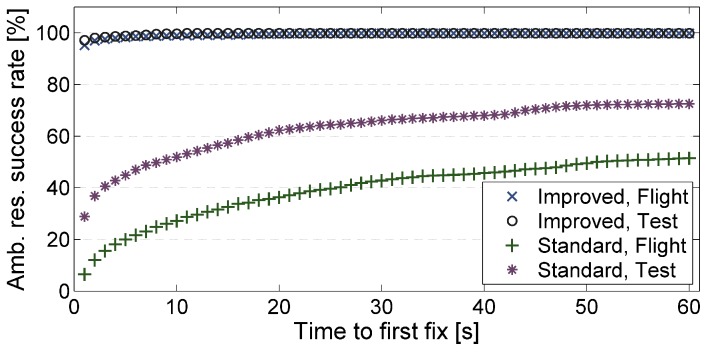
Ambiguity resolution (Amb. res.) success rates after a certain time, determined with a standard ambiguity resolution algorithm (standard) and our ambiguity resolution algorithm (improved) for flight test data (Flight) and test setup data (Test).

To answer the second question, the integer ambiguity estimation process was restarted in every epoch again, but now, the algorithm always proceeded for as many epochs as needed to resolve the ambiguities. In this way, each time to first fix could be measured. Figure 5 presents the percentage of cases in which the ambiguities could be resolved after a certain time. Here, also, a comparison is made between the standard and our improved ambiguity resolution algorithm. Accordingly, with our algorithm, the ambiguities could at least after 5 s be resolved in more than 99% of the cases during UAV flights. In contrast, with the standard algorithm, the ambiguities could after 5 s only be resolved in 20% of the cases during the UAV flights.

### 6.2. Accuracy

For the accuracy evaluation, the dataset “Test 2” is used in the following, which includes static and kinematic parts. During the kinematic part, the measurement setup was carried by two persons, trying to simulate typical UAV dynamics.

**Figure 6:**
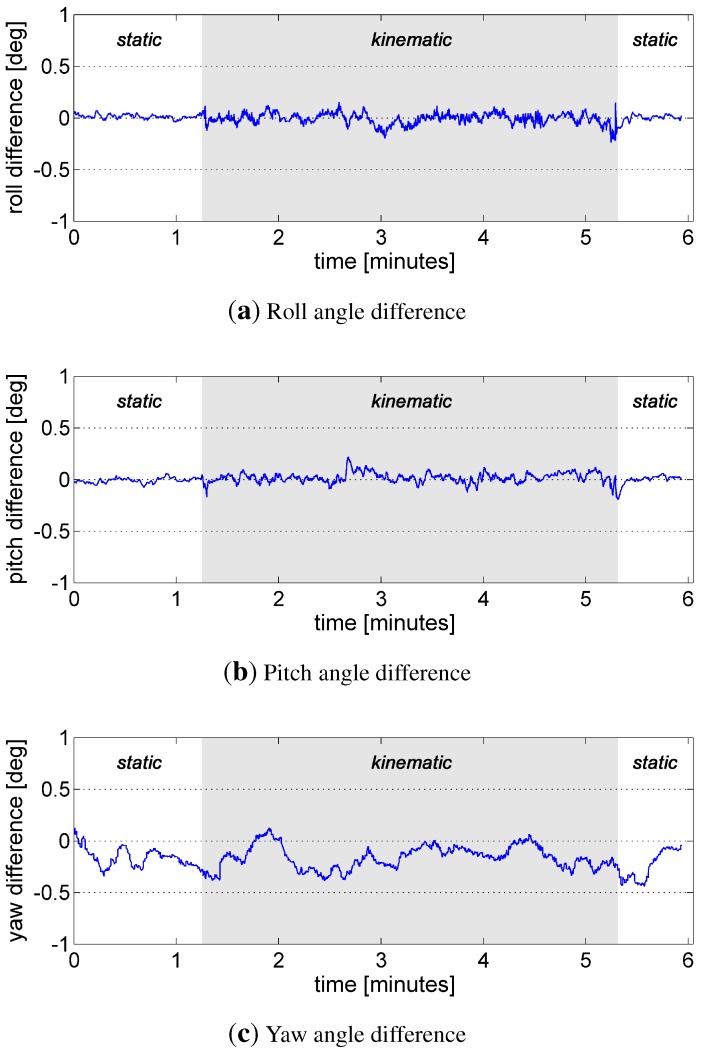
Difference between the reference Euler angles (Imar INS) and the Euler angles ((**a**): roll, (**b**): pitch, (**c**): yaw), which result from the Pos/IMU/Mag/Base integration of our system. The kinematic parts are marked with a gray background.

In Figure 6, the differences between the method described here and the reference attitude (Imar INS) are shown. It can be seen that the maximum difference is less than 0.5${}^{\circ }$ for all attitudes, where the roll and the pitch angle seem to be more accurate than the yaw angle. The mean, the root mean square (rms) values and the maximum deviations (max), which were determined on the basis of the shown differences, are presented in Table 3. The rms for the roll and the pitch are about 0.03${}^{\circ }$ in the static mode and 0.05${}^{\circ }$ in the kinematic mode. The rms for the yaw angle is about 0.2${}^{\circ }$ for a static and a kinematic mode, where the deviations of the yaw angle are mainly caused by the systematic errors of the GPS attitude baseline.

**Figure 7:**
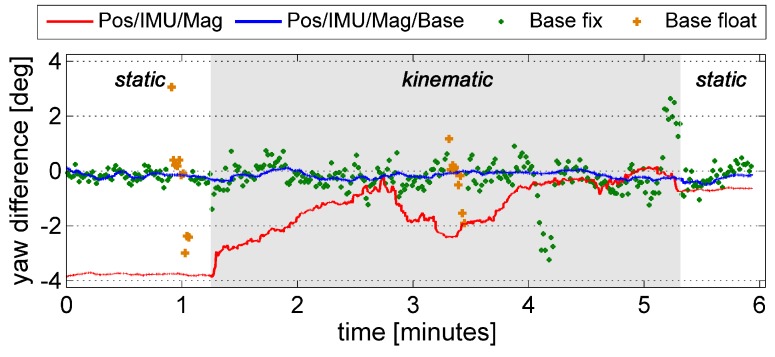
Difference of the GPS yaw angle (Base fix, Base float), the Pos/IMU/Mag/Base and the Pos/IMU/Mag yaw angle to the reference solution. The kinematic parts are marked with a gray background.

In Figure 7, the Pos/IMU/Mag/Base yaw angle differences from Figure 6 are compared to the GPS baseline attitude differences and the differences of the Pos/IMU/Mag solution. The maximum deviation of the Pos/IMU/Mag solution lies in the first static part of the measurement, where the yaw angle determination has mainly been influenced by the magnetic field observations. Even if these deviations are in the order of a few degrees, the solution still serves as good prior information for the ambiguity search. From experience, this is the same for UAV flights, where the deviations between the prior information and the GPS attitudes usually have a comparable magnitude to the deviations seen here. Due to the coupling with the GPS positions (we used RTK GPS positions), in combination with horizontal accelerations, the deviations decreased during the kinematic part of the measurement.

Although the ambiguities were held fixed for the GPS base solution (Figure 7), the cycle slip detection led to a re-initialization of the ambiguities in two epochs during this test. Furthermore, some GPS base outliers are also conspicuous (e.g., after *ca.* 4.2 min). However, the outliers do not deteriorate the attitudes from the Pos/IMU/Mag/Base integration.

**Table 3:** Results of an experiment with the test setup.

		Roll (${}^{\circ }$)	Pitch (${}^{\circ }$)	Yaw (${}^{\circ }$)
static	mean	0.009	−0.005	−0.188
	rms	0.027	0.032	0.221
	max	0.105	0.194	0.440
kinematic	mean	−0.007	0.018	−0.162
	rms	0.054	0.052	0.196
	max	0.237	0.221	0.388

## 7. Conclusions

In this paper, a direct georeferencing system has been presented, which is intended to provide highly accurate positions and attitudes (better than 5 cm and 0.5${}^{\circ }$) of a micro- and mini-sized UAV in real time. The paper has mainly been focused on the attitude determination with this system. For the attitude determination MEMS IMU readings, magnetic field observations, a GPS baseline and a GPS position measurement are integrated in a sixteen-state Kalman filter. To be able to resolve the ambiguities of the single-frequency GPS baseline reliably and instantaneously, the float solution has been improved. For the integer ambiguity estimation, the MLAMBDA method and the AFM are combined in a suitable way.

With a test measurement, where a navigation-grade INS served as a reference, it could be shown that the implemented algorithms allow for attitude accuracies of about 0.05${}^{\circ }$ for the roll and the pitch and 0.2${}^{\circ }$ for the yaw angle during kinematic applications. With the developed ambiguity resolution algorithms, the ambiguities of the single-frequency GPS attitude baseline could be resolved instantaneously in more than 90% of the epochs. It could also be shown that short-term losses of the GPS baseline can be bridged successfully by the inertial sensors without a deterioration of the attitude accuracy.

As a final conclusion, the use of a low-cost single-frequency GPS baseline can significantly improve the attitude determination of micro- and mini-sized UAVs, even in non-ideal GPS measurement environments.
